# Tuning Magnetoconductivity in LaMnO_3_ NPs through Cationic Vacancy Control

**DOI:** 10.3390/nano13101601

**Published:** 2023-05-10

**Authors:** Antonio Hernando, M. Luisa Ruiz-González, Omar Diaz, José M. Alonso, José L. Martínez, Andrés Ayuela, José M. González-Calbet, Raquel Cortés-Gil

**Affiliations:** 1 Departamento de Ingeniería, Universidad Antonio de Nebrija, Pirineos 55, 28940 Madrid, Spain; 2 Instituto de Magnetismo Aplicado, UCM-ADIF-CSIC, Las Rozas, 28230 Madrid, Spain; 3 IMDEA de Nanociencia Faraday 9, 28049 Madrid, Spain; 4 Donostia International Physics Centre, Manuel Lardizabal, Ibilbidea 4, 20018 San Sebastian, Spain; 5 Departamento de Química Inorgánica, Facultad de Químicas, Universidad Complutense de Madrid, 28040 Madrid, Spain; 6 Instituto de Ciencia de Materiales, CSIC, Sor Juana Inés de la Cruz s/n, 28049 Madrid, Spain; 7 ICTS-ELECMI-Centro Nacional de Microscopia Electrónica, Universidad Complutense de Madrid, 28040 Madrid, Spain

**Keywords:** nanoparticles, perovskite, manganites, cationic vacancies, magnetoconductivity, spin polarization

## Abstract

The inclusion of La-Mn vacancies in LaMnO_3_ nanoparticles leads to a noticeable change in conductivity behavior. The sample retains its overall insulator characteristic, with a typical thermal activation mechanism at high temperatures, but it presents high magnetoconductivity below 200 K. The activation energy decreases linearly with the square of the reduced magnetization and vanishes when the sample is magnetized at saturation. Therefore, it turns out that electron hopping between Mn^3+^ and Mn^4+^ largely contributes to the conductivity below the Curie temperature. The influence of the applied magnetic field on conductivity also supports the hypothesis of hopping contribution, and the electric behavior can be explained as being due to an increase in the hopping probability via spin alignment.

## 1. Introduction

**Figure 1:**
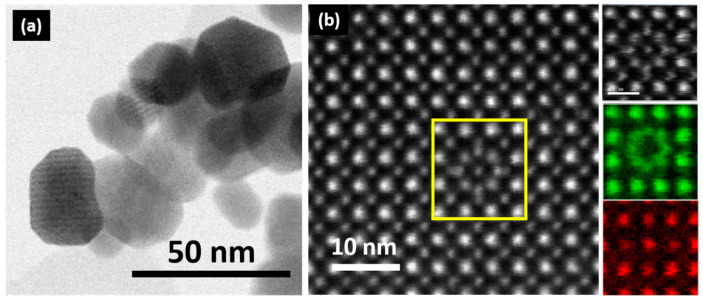
(**a**) Low-magnification TEM image corresponding to La_0.93_Mn_0.93_MnO_3_ NPs. (**b**) HAADF atomic-resolved detail of a La_0.93_Mn_0.93_MnO_3_ NP and the chemical maps, on the right hand of the image (green indicates La and red indicates Mn), of the simultaneously acquired HAADF image of the area indicated by the yellow box.

Manganese perovskite-related oxides are well-known functional materials due to their strongly correlated electronic and magnetic properties, among other features. Among these oxides, LaMnO_3_ perovskite is, in spite of its simple composition, a complex system that can accommodate compositional variations in the form of cationic vacancies. Initial studies showed that, as a function of the synthesis conditions, additional oxygen content (δ) could be accommodated to formulate LaMnO_3+δ_. Nevertheless, neutron diffraction [1,2,3,4,5] studies discarded the presence of interstitial oxygen and unveiled the presence of vacancies in both cationic sublattices, leading to the La_1-t_Mn_1-t_O_3_ (t = δ/(δ + 3)) formulation. Consequently, La_1-t_Mn_1-t_O_3_ (t = δ/(δ + 3)) more appropriately describes the stoichiometry of this system, although, for simplicity reasons, the previous formulation LaMnO_3+δ_ is frequently used. The occurrence of cationic vacancies is a critical parameter since Mn^3+^ and Mn^4+^ coexist in La_1-t_Mn_1-t_O_3_, leading to alternative diffusion paths and unconventional magnetic and transport properties. Furthermore, if the particle size is reduced down to the nanometric scale, novel and outstanding features appear [6,7,8,9]. While the crystallochemical parameters determine the properties of the bulk material, the size and morphology effects of nanoparticles (NPs) are responsible for these emerging properties. Accordingly, by using appropriate synthesis strategies, we have recently shown [10] that homogeneous LaMnO_3+δ_ NPs, with an average size of 20 nm (Figure 1a), significantly modify their microstructure and magnetotransport properties as a function of the cationic vacancy concentration. For δ = 0, the atomically resolved Scanning Transmission Electron Microscopy (STEM)–High-Angle Annular Dark Field (HAADF)–Electron Energy Loss Spectroscopy (EELS) studies showed that the typical atomic distribution of La and Mn atoms is in agreement with the perovskite lattice, being 3+ in the Mn oxidation state (Figure 1b). Nevertheless, for δ = 0.23, the NPs exhibit defects that involve local displacements of La atoms from their normal sites (Figure 1b) and the coexistence of Mn^3+^ and Mn^4+^. Similar to the bulk material, LaMnO_3_ NPs, i.e., with only Mn^3+^, show insulator behavior without traces of magnetoresistance, but their ground state changes from antiferromagnetic (AFM) to ferromagnetic (FM) when the particle size is reduced. On the other hand, for δ = 0.23, i.e., La_0.93_Mn_0.93_O_3_ NPs, compositional variations are accommodated by cationic vacancies, which seem to be responsible for the weaker FM interactions promoting the emergence of magnetoresistance in the insulator material. In similar bulk compositions, the presence of Mn^4+^ favors the emergence of FM interaction via Mn^3+^-O^2−^-Mn^4+^ double-exchange interactions; however, in these NPs, the FM interaction decreases in spite of the presence of 45% of Mn^4+^. This behavior seems to be due to the displacement of some La atoms around their normal sites, which introduces some restrictions in the double-exchange Mn^3+^-O^2−^-Mn^4+^ interactions. Based on these facts, the origin of magnetotransport should be governed by a super-exchange mechanism. To shed light on these findings, in this study, we analyzed the temperature dependence of conductivity on magnetization, leading to an exponential law in which the activation energy decreases as a function of magnetization. This characteristic accounts for the presence of magnetoresistance and points out that the energy barrier required to activate carriers is completely erased by saturating the spin polarization.

## 2. Materials and Methods

La_0.93_Mn_0.93_O_3_ NPs were prepared based on the molten salt synthesis method, as described in [10], by mixing corresponding metal nitrate precursors with molten salt KNO_3_. The molar ratio of reactants to molten salt was 1:5 using ethanol as the dispersion agent. After being dried under vacuum overnight at 40 °C, the powder was heated at 650 °C for 2 h and then quenched at room temperature. The product was washed and centrifuged several times with distilled water. After washing, the sample was dried at 50 °C for 24 h. LaMnO_3_ NPs were obtained via topotactic reduction of La_0.93_Mn_0.93_O_3_ in a Cahn D-200 electrobalance equipped with a furnace at 350 °C under a H_2_ (200 mbar) and He (300 mbar) atmosphere. On the other hand, the starting bulk material was synthesized using the ceramic method. Stoichiometric amounts of La_2_O_3_ and MnO_2_ were homogenized and milled in an agate mill and heated at 1400 °C for 100 h, with several intermediate grindings. After quenching in liquid nitrogen, a single phase with La_0.93_Mn_0.93_O_3_ composition was obtained. This sample was also reduced in the electrobalance under a H_2_/He atmosphere at 360 °C in order to obtain bulk LaMnO_3._

Cation compositional analysis was determined in all samples by means of electron probe microanalysis (EPMA) using a JEOL JXA-8900 microscope and analyzed around 20 areas of 1–5 mm. X-ray diffraction (XRD) was performed using a PANalytical X’pert PRO diffractometer operating with Cu Kα1 radiation in the Bragg–Brentano geometry at room temperature.

High-resolution TEM was performed using a JEOL JEM 300FEG electron microscope equipped with an energy-dispersive X-ray spectroscopy (EDS) microanalysis system (Oxford INCA). Atomic-resolved STEM-EELS characterization was performed using a probe spherical aberration-corrected microscope JEOL JSM-ARM200F (Cold Emission Gun). The microscope was operated at 120 kV in order to minimize the damage of the samples. Inner and outer collection semi-angles of 68 and 280 mrad were set for the acquisition of atomically resolved high-angle annular dark-field (HAADF) images. The microscope was equipped with a GIF-QuantumERTM spectrometer, used for the EELS experiments (with a collection semi-angle of 18 mrad and a convergence semi-angle of 20.3 mrad). Details of the analytical procedures are described in [10].

Magnetization was measured using a SQUID magnetometer from Quantum Design with a magnetic field of up to 5 T in a temperature range from 2 to 200 K. The resistivity measurements were performed using a Physical Properties Measurement System (PPMS) from Quantum Design with a temperature range from 2 to 400 K and an external magnetic field of up to 9 T.

## 3. Results and Discussion

**Figure 2:**
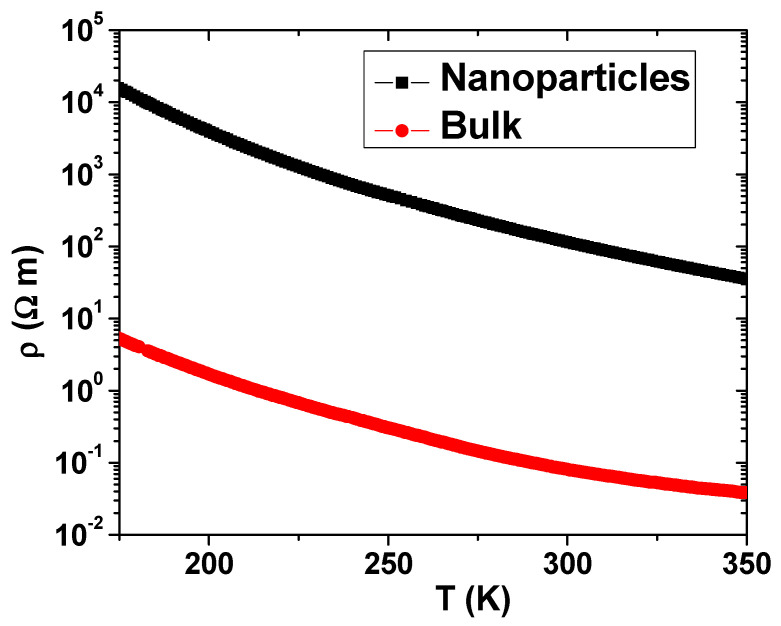
Temperature dependence of resistivity for the LaMnO_3_ NP and bulk ceramic samples in the high temperature range above the Curie temperature in which the exponential behavior with constant activation energy holds.

In this report, we focus our analysis on the electric conductivity and magnetoconductivity of La_0.93_Mn_0.93_O_3_ and LaMnO_3_ NPs. As described in reference [10], bulk LaMnO_3_, in which all Mn atoms are Mn^3+^ ions, behaves as an insulator antiferromagnet; however, when its size is sufficiently small (diameter below 30 nm), it becomes ferromagnetic. The differences in the electrical resistance behavior between the bulk and LaMnO_3_ NP samples lie in the large contribution coming from the contact between contiguous NPs. Even though our measurements of the NPs were performed on pellets obtained under 6 ton/cm^2^ pressure, the resistivity values of the NPs are three orders of magnitude higher than those corresponding to the bulk counterpart, as shown in Figure 2. However, the resistivity in both cases fits well, at high temperatures, to an exponential thermal activation law with the same T_0_ = 2606 K or an activation energy of 0.224 eV, as derived from the curves shown in Figure 2. It is worth noting that this value is in agreement with the value reported by Mahendiran et al. [11] for polycrystalline samples of the same composition. It can be concluded that the conductivity of LaMnO_3_ is independent of the sample size and is caused by thermal activation of carriers with an activation energy close to 0.224 eV. When these LaMnO_3_ NPs contain 45 at% of Mn^4+^ (which, based on the electro-neutrality restrain, corresponds to 7 at% of La-Mn vacancies, i.e., La_0.93_Mn_0.93_O_3_), they retain their insulator characteristic, and the high-temperature resistivity fits an exponential behavior but with decreasing T_0_ to 1600 K or decreasing activation energy to 0.138 eV. 

Regarding magnetic measurements, it is important to consider that both LaMnO_3_ and La_0.93_Mn_0.93_O_3_ NPs, with a similar size distribution centered at 25 nm, are ferromagnetic with the Curie temperature close to 220 K. The ferromagnetism of LaMnO_3_ should stem from the super-exchange interactions between Mn^3+^ ions, whereas that of La_0.93_Mn_0.93_O_3_ could also be originated from Mn^3+^-O^2−^-Mn^4+^ double-exchange interactions. However, the measured resistivity of the two samples is at 400 K, which is one order of magnitude higher for LaMnO_3_, while this difference increases to three orders of magnitude at 10 K. Even though these differences in the activation energy of both samples are remarkable, the absolute values of conductivity are several orders of magnitude lower than those characteristic of metals, and, therefore, both samples can be considered insulators. The insulator characteristic points out the lack of double exchange.

As it is well known that when the ferromagnetic order is originated from double exchange, the delocalized electron hopping between adjacent Mn ions form a conduction band that gives rise, as concerning conductivity, to the metallic behavior of the sample. Double exchange appearing in the samples in which Mn^3+^ and Mn^4+^ ions coexist is a consequence of the combination of two facts. First, the electron energy decreases with delocalization and, secondly, spin alignment of adjacent ions favors the hopping. On the other hand, if the cause of ferromagnetism is due to super-exchange interactions, the motion of electrons should be generally driven by thermal excitation, as is the case of LaMnO_3_ in which all Mn ions are Mn^3+^. However, in the samples in which the main exchange contribution comes from super-exchange interactions but contain both Mn^3+^ and Mn^4+^ ions, the electron motion can take place via either thermal excitation or hopping between adjacent ions with different valence. The last contribution, being dependent on the spin alignment, is expected to be dependent on the average magnetization, i.e., on the applied magnetic field and vanishes at temperatures above the ferromagnetic order. Therefore, the electron hopping contribution should be associated with the dependence of conductivity on the applied magnetic field.

From the presence of high magnetoresistance effects in La_0.93_Mn_0.93_O_3_, it shows the existence of a remarkable contribution of spin order to conductivity. By comparing with the electrotransport behavior of LaMnO_3_ NPs, in which magnetoresistance effects are not observable, it seems reasonable to link this difference to the presence of Mn^4+^ atoms. In this report, we show that Mn^3+^-O^2−^-Mn^4+^ electron–hole hopping is extremely dependent on the sample magnetization and largely contributes to both conductivity and magnetoconductivity at temperatures below 200 K, i.e., below its Curie temperature. In fact, it can be concluded that the activation energy depends on magnetization.

The origin of conductivity in insulator manganites has been proposed to be due to different microscopic mechanisms [12,13,14,15,16]. Variable-range hopping [17] semiconductor behavior, and small polaron [18] model have been invoked by different groups using their corresponding expressions for their temperature dependence. However, we aimed to analyze the experimental results while assuming a simple exponential dependence, such as that given by Equation (1), but where T* is considered to be a function of magnetization and, thereby, of the applied magnetic field. Our target consists of finding, from the experimental results, how the activation energy depends on magnetization.

**Figure 3:**
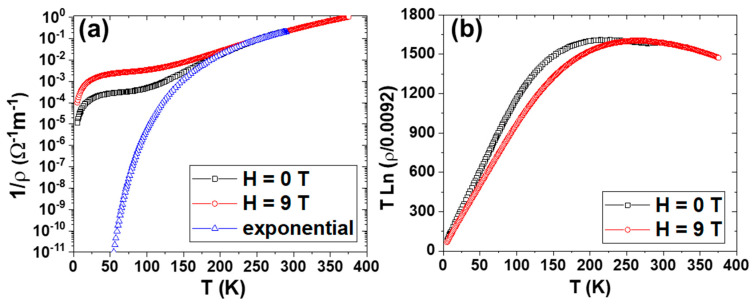
(**a**) Temperature dependence of the inverse of resistivity of La_0.93_Mn_0.93_MnO_3_ NPs under 0 and 9 T. The parameters considered here are T* (T→∞) = 1600 K and ρ_∞_ = 0.008 Ωm. The exponential fitting is included. (**b**) Temperature dependence of T* of La_0.93_Mn_0.93_MnO_3_ NPs under 0 and 9 T.

Figure 3a illustrates conductivity (or the inverse of resistivity) as a function of T for La_0.93_Mn_0.93_O_3_ NPs under different magnetic fields. The temperature dependence of conductivity, measured at zero applied field, deviates from its high-temperature exponential behavior just at the temperature range (below 200 K) at which the NPs start to be magnetic; this deviation increases with spontaneous magnetization. At T = 100 K, the conductivity is five orders of magnitude smaller than the value corresponding to the thermal excitation rate governed by the exponential fitting. It is obvious that, notwithstanding the great complexity of possible conduction mechanisms, such changes of several orders of magnitude in the conductivity values should be mainly due to changes in the density of carriers. Consequently, the profile shown in Figure 3a roughly depicts the number of available carriers as a function of temperature.

It is interesting to observe that the conductivity under the applied magnetic field seems to be enhanced only for temperatures below the Curie point. Furthermore, the effect of the applied magnetic field is also associated with a clear conductivity enhancement. In other words, the magnetic order promotes the hopping processes induced by the applied magnetic field.

At temperatures above the Curie temperature, the effect of the magnetic order on the number of carriers disappears, and the resistivity of La_0.93_Mn_0.93_O_3_ NPs shows the same temperature dependence observed in LaMnO_3_ NPs, i.e.,

(1) $$\rho_{th}=\rho_{\infty }e^{\frac{T^{*}}{T}}$$

However, as *T* decreases, below the Curie temperature, the number of carriers becomes much larger than that expected for T* = 1600 K. This behavior can be explained as a result of a decrease in T*, which can be obtained from the experimental values and plotted in Figure 3a, according to the following expression derived from (1):

(2) $$T^{*}\left(T\right)=Tln\frac{\rho_{th}}{\rho_{\infty }}$$

Figure 3b shows the temperature dependence of T* for zero and 9 *T* applied fields. It is worth noting that the function T*(*T*) obtained experimentally can be well-fitted to the classical magnetoconductivity dependence on the square of the reduced magnetization, according to the following relationship:

(3) $$T^{*}\left(T\right)=T^{*}\left(\infty \right)(1-{\left(\frac{M_{s}\left(T\right)}{M_{s}\left(0\right)}\right)}^{2})$$

This dependence is the value corresponding to the hopping probability dependence of the relative spin orientation of Mn^3+^ and Mn^4+^ adjacent ions.

**Figure 4:**
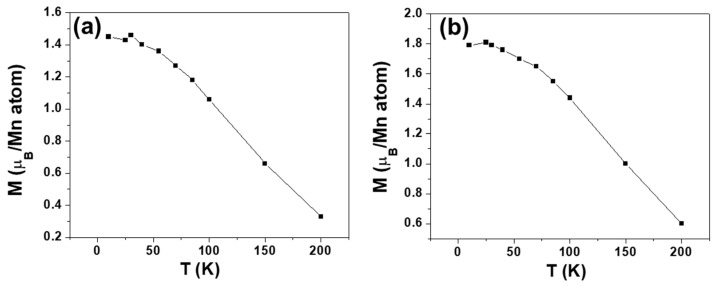
Magnetization as a function of temperature of La_0.93_Mn_0.93_MnO_3_ NPs at (**a**) 2 T and (**b**) 5 T.

Figure 4 illustrates the temperature dependence of magnetization when the sample undergoes an applied field of 2 and 5 T.

**Figure 5:**
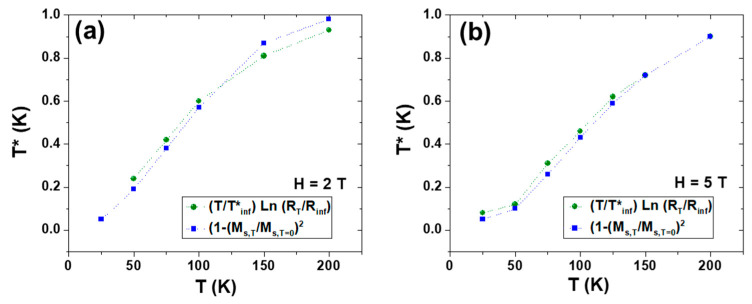
Experimental thermal temperature dependence of T* of La_0.93_Mn_0.93_MnO_3_ NPs obtained from Equation (2). The agreement with the T* values calculated according to Equation (3), which are also plotted in the figure, becomes noticeable. (**a**) Applied field of 2 T. (**b**) Applied field of 5 T.

From the curves plotted in Figure 4, it is possible to obtain the corresponding T* at any T using Equation (3) (Figure 5) and to observe that these values coincide with those obtained from the experimental resistivity obtained from Equation (2) and plotted in Figure 3b. 

In conclusion, the conductivity behavior of the sample is governed by an activation energy that obeys Equation (3). Moreover, it also points to the fact that the activation energy for the electron–hole hopping process, which is available in the presence of Mn^3+^ and Mn^4+^ ions, decreases linearly with the square of the reduced magnetization and consequently vanishes when saturation is reached. The analyzed behavior requires the presence of Mn^3+^ and Mn^4+^ ions but also the absence of long-range double exchange; otherwise, the sample would be metallic. 

Our experimental data were then checked against some theoretical works. The lack of long-range double-exchange interactions between Mn^3+^ and Mn^4+^ when adding Mn^4+^ ions in LaMnO_3_ NPs requires further discussion. In addition, magnetization per Mn atom decreases and is ascribed to Mn ions near the vacancies and the surface of NPs. Furthermore, in contrary to the bulk system, the accommodation of cationic vacancies at the nanoscale leads to interstitial defects, which hinders the required Mn^3+^-O^2−^-Mn^4+^ overlapping in La_0.93_Mn_0.93_O_3_ NPs to lead to the ferromagnetic and metallic behavior of double-exchange interactions. 

Experimentally, it seems that surface effects also lead to the large observed differences. We herein consider the magnetic interaction between Mn^3+^ and Mn^4+^ ions within mixed-valence manganites included in the large class of magnetic semiconductors. We comment on the trends based on a model that has magnetic ions interacting with mobile carriers as in diluted magnetic semiconductors, following Coey et al. [19]. Theoretically, the decrease in magnetization per atom could be explained by considering the polarons associated with defects. From an atomistic point of view, the equivalence between Mn^3+^ + h^+^ and Mn^4+^ could explain the decrease, but this situation is associated with a decrease in the Curie temperature due to Mn-O-Mn clustering when there is hole localization [20,21]. Since the above experiments showed almost the same Curie temperature, this possibility could be ruled out, and we are, thus, left with the role of surface effects.

The surface effects, regarding double-exchange magnetic interaction, are next described in more detail. The d-Mn levels interact ferromagnetically when they are aligned in energy. However, the surface of NPs modifies the crystalline field around Mn ions so that they do not interact ferromagnetically through double exchange and prefer a more superexchange-like magnetic order. This interpretation agrees with the calculations for Mn-doped semiconductors in nanostructures where the surface modifies the crystalline field and the hole-mediated ferromagnetism disappears [22].

## 4. Conclusions

In summary, the electrical conductivity in La_0.93_Mn_0.93_O_3_ can be carried out through two different channels: (a) pumping of electrons to the conduction band with an activation energy in the order of 0.14 eV, and (b) hopping of electrons from Mn^3+^ and Mn^4+^ ions. Since the latter mechanism requires the parallelism of the magnetic moment of both ions, it becomes present only below the Curie temperature, with an activation energy dependent on the square of the reduced magnetization.

In the sample analyzed here, double exchange is blocked; however, the magnetic field applied to measure the conductivity drives the electron–hole hopping. Therefore, reciprocally, the electric current probably tends to align the spins of two adjacent ions, an interesting suggestion to be investigated in future work.

## Data Availability

All data generated or analyzed during this study are included in this published article.
